# Preliminary study on the predictive value of the vasoactive-inotropic score for the prognosis of neonatal congenital diaphragmatic hernia

**DOI:** 10.1186/s12893-023-01970-3

**Published:** 2023-03-29

**Authors:** Chao Liu, Jingna Li, Yandong Wei, Ying Wang, Hui Zhang, Lishuang Ma

**Affiliations:** 1 Department of Pediatric and Neonatal Surgery, Capital Institute of Pediatrics, Yabao Road #2, 100020 Beijing, China; 2 Department of Cardiac Surgery, Capital Institute of Pediatrics, Beijing, China

**Keywords:** Hernia, Diaphragm, Neonate, Vasoactive-inotropic score, Prognosis

## Abstract

**Background:**

No study has reported on the relationship between the vasoactive-inotropic score (VIS) and the prognosis of neonates with a severe congenital diaphragmatic hernia (CDH). This study aimed to identify potential risk factors for mortality in patients with CDH. We calculated the VIS based on the vasoactive drugs used during the perioperative period to investigate the relationship between VIS and infant prognosis.

**Methods:**

We retrospectively analyzed the clinical data of 75 neonates with CDH who were treated at our center between January 2016 and October 2021. We calculated the maximum and mean VIS during the first 24 h of hospitalization (hosVIS [24max] and hosVIS [24mean], respectively) and after surgery (postVIS [24max] and postVIS [24mean], respectively). The relationship between the VIS and the prognosis of neonates with CDH was analyzed using a receiver operating characteristic (ROC) curve, *t*-test, chi-square test, rank-sum test, and logistic regression analysis.

**Results:**

In total, 75 participants with CDH were included in the study. The chance of survival was 80%. Our results showed that hosVIS (24max) was an accurate predictor of prognosis (area under the ROC curve = 0.925, *p* = 0.007). The calculated optimal critical value of hosVIS (24max) for predicting a poor prognosis was 17 (J = 0.75). Multivariate analysis revealed that hosVIS (24max) was an independent risk factor for death in neonates with CDH.

**Conclusion:**

In neonates with CDH, a higher VIS, especially hosVIS (24max), suggests worsened cardiac function, a more severe condition, and a higher risk of death. The rising VIS score in infants prompts physicians to implement more aggressive treatment to improve cardiovascular function.

## Background

Congenital diaphragmatic hernia (CDH) is a developmental defect in the diaphragm, leading to the protrusion of abdominal organs into the thoracic cavity, causing a series of pathophysiological changes. The incidence of CDH ranges from approximately 1/5000 to 1/2000 births [1]. Pulmonary hypoplasia and persistent pulmonary hypertension (PPHN) are the primary causes of death [1–4]. In addition to treating pulmonary hypertension, vasoactive drugs are regularly utilized to improve circulatory function in neonates with CDH because PPHN and PH can cause cardiac dysfunction [3]. The vasoactive-inotropic score (VIS), which reflects the postoperative severity of congenital heart diseases in pediatric patients, helps clinicians evaluate the patient’s condition and predict prognosis [5, 6]. A high VIS has been linked to poor outcomes [5, 6]. Furthermore, the VIS contains vasoactive and positive inotropic drugs usually administered to neonates with CDH. Consequently, VIS can not only reflect the severity of PPHN in neonates, but also the difficulty of treating pulmonary hypertension. Therefore, VIS may be used to establish the degree of cardiovascular medication support, assess the severity of disease, and act as an early predictor of mortality in infants with CDH. In the operating room and intensive care unit, inotropes and vasopressors are routinely used to maintain blood pressure. Death rates increase when large doses of vasoactive drugs are necessary, and a high VIS has been linked to poor outcomes [5–7]. However, no study has reported on the relationship between the VIS and the prognosis of neonates with CDH. Therefore, we aimed to determine whether a higher VIS indicates a greater mortality risk.

## Methods

### Ethics statements

This study protocol was approved by the Ethics Committee of the Capital Institute of Pediatrics (approval number: DWLL2019005). Written informed consent was obtained from the parents of patients with CDH before the study.

### Study design and patients

The records of 89 neonates with CDH who were treated in our center between January 2016 and October 2021 were reviewed. Inclusion criteria were as follows: neonates were diagnosed antenatally with CDH and were confirmed by chest X-ray after birth; neonates with respiratory distress who required intubation and ventilation immediately after birth; infants admitted within 24 h after birth; and patients who underwent repair surgery. Exclusion criteria were as follows: neonates diagnosed with esophageal hiatal hernias after birth (*n* = 4); neonates who showed signs of CDH other than postnatal dyspnea > 24 h after birth (n = 6); and neonates who died without undergoing surgery before clinical stability (n = 4).

### Neonatal protocol

All neonates were diagnosed through prenatal ultrasonography. After birth, ultrasonography and chest X-ray scans confirmed the diagnosis of congenital diaphragmatic hernia (CDH). In the delivery room, newborns were intubated and given a nasogastric tube shortly after birth; then, they were transferred to the neonatal intensive care unit. All infants were given gentle ventilation, allowed to have permissive hypercapnia, had invasive blood pressure measurements taken, and had continuous monitoring of their arterial oxygen saturation. High-frequency oscillation ventilation was performed only when it was absolutely essential. Within the first 24 h after birth, echocardiography was the best modality for excluding the presence of cardiac anomalies, assessing heart function. The severity of pulmonary hypertension was evaluated on echocardiography based on the degree of TR and the direction of the PDA shunt. Transverse regurgitation was rated by echocardiography as non-existent, mild, moderate, and severe. PDA shunts were classified as either left-to-right, bidirectional, or right-to-left according to the direction of blood flow [8].

It was suggested that the average arterial pressure was maintained at 40–45 mmHg (1 mmHg = 0.133 kPa), the capillary filling time at 3 S, the lactic acid content in arterial blood gas at 3mmol / L, and the urine volume at > 1ml / kg / h. When the measured mean arterial pressure was lower than the relevant fetal age level and the abovementioned indicators did not meet the criteria, hypoperfusion was diagnosed. The initial action was a process to increase volume. If blood pressure and tissue perfusion could not be restored, then vasoactive drugs like dopamine, dobutamine, and epinephrine needed to be given as soon as possible. It was common practice to combine several medications. The drug’s usage and dosage are shown in the Table 1. Our treatment did not include fetal endoscopic tracheal blockage or extracorporeal membrane oxygenation.

**Table 1:** Vasoactive medications commonly used in CDH

Drug	Route	Units	Initial dose	Maintenance Dose range
Dopamine	IV	µg/kg/min	3 to 5	10 to 15 (usually 10)
Dobutamine	IV	µg/kg/min	3 to 5	10
Epinephrine	IV	µg/kg/min	0.05 to 0.1	0.05 to 1
Norepinephrine	IV	µg/kg/min	0.03 to 0.1	0.05 to 1
Milrinone	IV	µg/kg/min	25-75ug/kg iv. >30 min	0.25 to 0.75

Abbreviations: IV, Intravenous infusion; µg/kg/min, micrograms per kilogram of body weight per minute; ug/kg, micrograms per kilogram of body weight

### Outcome measure and data collection

The primary outcome measure was the survival before discharge. The demographic and clinical data analyzed in this study included gestational age (GA) at birth, sex, birth weight, GA at prenatal diagnosis, side of defect, the observed-to-expected lung-to-head ratio (O/E LHR) by fetal ultrasonography, presence of liver herniation into the thoracic cavity, the severity of PPHN [8], time and surgery approach (open or thoracoscopic surgery), maximum VIS during the first 24 h of hospitalization (hosVIS [24max]), mean VIS during the first 24 h of hospitalization (hosVIS [24mean]). Maximum VIS during the first postoperative 24 h (postVIS [24max]), mean VIS during the first postoperative 24 h (postVIS [24mean]).

In left-sided CDH, an O/E LHR < 25% indicates a poor result; in right-sided CDH, an O/E LHR < 45% may suggest a poor outcome [9, 10]. We grouped patients without pulmonary hypertension (PH) and mild PH into one group and patients with moderate to severe PPHN into another group [10, 11]. The average value of VIS was determined based on the actual survival time if the infant died within 24 h of birth or within 24 h after surgery. Preoperative peak lactate level, peak lactate level on the first day postoperatively, the average duration of mechanical ventilation, and mortality (stopping treatment halfway was considered death).

### Calculation of VIS

The VIS was calculated using the following equation [12]: VIS = [dopamine dose (µg/kg/min)] + [dobutamine dose (µg/kg/min)] + [10×milrinone dose (µg/kg/min)] + [100×epinephrine dose (µg/kg/min)] + [100×norepinephrine dose (µg/kg/min)] + [10 000×vasopressin (U/kg/min)].

All infants were admitted to the surgical neonatal intensive care unit. When an infant showed signs of low perfusion, interventions including volume expansion and the administration of vasoactive drugs were performed as soon as possible. Hourly VIS values were recorded, and hosVIS (24mean), postVIS (24mean), hosVIS (24max), and postVIS (24max) were calculated.

### Statistical analysis

The sample size was determined by the inclusion analysis of cases admitted to the center between January 2016 and October 2021 that satisfied the inclusion criteria. Continuous data with a normal distribution are expressed as mean ± standard deviation, while those with a non-normal distribution are expressed as median and interquartile range. Data were compared between the groups using the unpaired t-test or rank-sum test. Categorical data are expressed as percentages. Data were also compared using a four-fold table chi-square or rank-sum test. A receiver operating characteristic (ROC) curve was used to evaluate the predictive power of the VIS. The prediction cut-off value of each indicator was taken when the Youden index was at a maximum. A multivariate logistic regression model was used to identify independent risk factors for severe neonatal CDH. Statistical analyses were performed using SPSS, version 25.0 (IBM Corp., Armonk, NY, USA). Statistical significance was set at *p* < 0.05.

## Results

### Clinical data

Of the 75 neonates enrolled, 41 (54.7%) were male. There were 61 (81.3%) and 14 (18.7%) cases of left-sided and right-sided CDH, respectively. The mean GA at prenatal diagnosis was 24.7 ± 3.2 weeks (range: 20–37 weeks) and the mean GA at birth was 37.3 ± 1.2 weeks (29.29–40.86 weeks). The liver was intrathoracic in 23 (30.7%) neonates. Inotropes and vasopressors were used in 62 (82.7%) and 69 (92%) neonates preoperatively and postoperatively, respectively. All patients underwent surgery: 46 (61.3%) and 29 (38.7%) neonates underwent thoracoscopic and open surgery, respectively. The mean timing of operation, which refers to the period from birth to surgery, was 25.37 ± 2.2 h. The average ventilation time was 176.81 ± 2.4 h. Survival before discharge was 80% (n = 60). Table 2 shows the baseline characteristics of CDH patients.

**Table 2:** Baseline characteristics of CDH patients

Variables	Numbers (n = 75)
Gestational age (weeks)	37.3 ± 1.2
Male sex (n and %)	
Male	41(54.7%)
Female	34(45.3%)
Birth weight (g)	
GA at diagnosis (weeks)	2984.6 ± 239.2
Side of defect (n and %)	24.7 ± 3.2
Left	61(81.3%)
Right	14(18.7%)
Liver herniation (n and %)	23(30.7%)
Chromosomal anomaly (n and %)	6(8%)
O/E LHR < 25%(left) (n and %) or O/E LHR < 45%(right)	19(25.33%)
Severity of PPHN (n and %)	
Trivial and mild	28(37.3%)
Moderate and severe	47(62.7%)
Mean timing of surgery (hours)	25.37 ± 2.2
Surgery approach (n and %)	
Thoracoscopic	46(61.3%)
Open	29(38.7%)
Inotropes and vasopressors used (n and %)	
Preoperative	62(82.7%)
Postoperative	69(92%)
Average ventilation time(hours)	176.81 ± 2.4
Survival before discharge (n and %)	60(80%)

Abbreviations: CDH, congenital diaphragmatic hernia; GA, gestational age; PPHN, persistent pulmonary hypertension

### Comparison of VIS and other parameters

In Table 3, continuous variables were converted to categorical variables. The parameters with statistically significant difference between the death group and the survival group (p < 0.05) are shown in Table 3. According to the vasoactive medications, the death group had significantly higher hosVIS (24max), hosVIS (24mean), postVIS (24max), and postVIS (24mean) than the survival group (all *p* < 0.05). The peak lactate levels preoperatively and postoperatively were higher in the death group than in the survival group.

**Table 3:** Comparison of main outcomes based on survival before discharge

	Survival at discharge n = 60	Died n = 15	*z/t/X*^*2*^ value	P-value
Gestational age (weeks)	37.71 (37, 38.71)	37 (35.57, 39)	1.022	0.307
Male sex (%)	36 (60.00)	55 (33.33)	3.443	0.064
Birth weight (g)	3000 (2655, 3350)	2920 (2520, 3470)	0.146	0.884
GA at diagnosis (weeks)	26.53 ± 4.75	23.93 ± 2.49	2.925	0.005
Right-sided defect (n and %)	9 (15%)	5 (33.33%)	1.586	0.208
Liver herniation (%)	14 (23.33)	9 (60.00)	5.961	0.015
O/E LHR < 25%(left) (n and %) or O/E LHR < 45%(right)	11(18.3%)	9(53.3%)	6.031	0.014
Chromosomal anomaly (n and %)	3 (5.00)	3 (20.00)	3.623	0.090
Moderate and severe PPHN (n and %)	33 (53.33)	15 (100)	-	0.002
Timing of surgery (hours)	26 (24, 31.75)	16 (12, 26)	3.616	< 0.001
Thoracoscopic surgery (n and %)	39 (65.00)	7 (46.67)	1.701	0.192
HosVIS (24max)	5 (0, 10)	16 (13, 24)	-5.784	< 0.001
HosVIS (24mean)	3.95 (0, 6.5)	10.6 (7, 17.4)	-4.444	< 0.001
PostVIS (24max)	8.43 (0, 12)	27.73 (18,56)	-4.970	< 0.001
PostVIS (24mean)	7.75 (5, 11.8)	23.7 (14.4, 30.2)	-4.657	< 0.001
Preoperative peak lactate level (mmol/L)	2.4 (1.9, 3.4)	4.6 (3.9, 5.6)	-4.061	< 0.001
Postoperative peak lactate level on the 1st day (mmol/L)	2.4 (1.7, 3.92)	8.9 (7.3, 11)	-5.002	< 0.001

Abbreviations: GA, gestational age; PPHN, persistent pulmonary hypertension; VIS, vasoactive-inotropic score; hosVIS (24max), maximum vasoactive-inotropic score during the first 24 h of hospitalization; hosVIS (24mean), vasoactive-inotropic score during the first 24 h of hospitalization; postVIS (24max), maximum vasoactive-inotropic score during the first postoperative 24 h; postVIS (24mean), vasoactive-inotropic score during the first postoperative 24 h

### Predictive value of the VIS

**Fig. 1:**
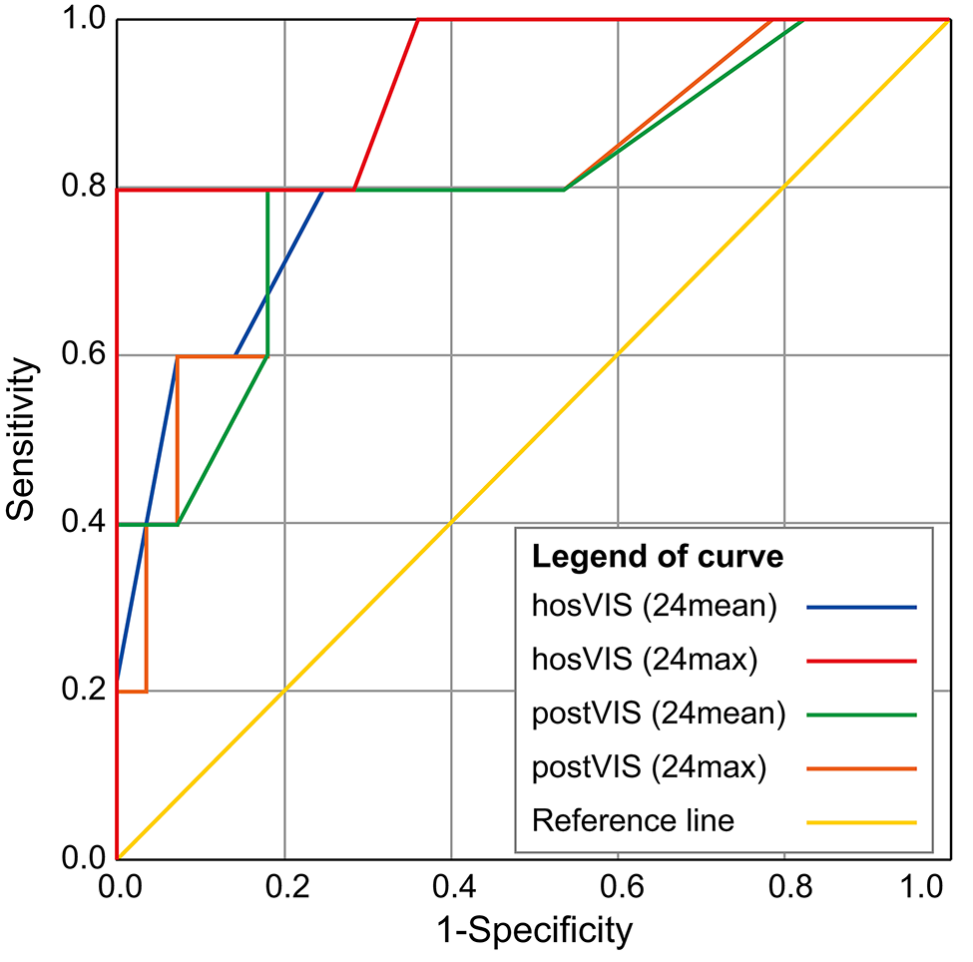
The receiver operating characteristic curve predicts the discriminating ability of vasoactive inotropic scores (VISs) in neonates with congenital diaphragmatic hernia. hosVIS (24max), maximum vasoactive inotropic score during the first 24 h of hospitalization; hosVIS (24mean), mean vasoactive inotropic score during the first 24 h of hospitalization ROC curve of vasoactive-inotropic score (VIS) in predicting death of infants with congnital diaphragmatic hernia: The AUROC values of those four VIS scores were relatively high, suggesting that they were more accurate predictors of prognosis in neonates with CDH. In Particular, the hosVIS (24max) had the highest accuracy (AUROC = 0.925, P = 0.007)

Since the VIS was related, we used an ROC curve to assess the predictive value of the factor. The relationship between VIS and the prognosis of patients was determined using the ROC curve and area under the ROC curve (AUROC) (Fig. 1). AUROC values of the four VIS were high, especially hosVIS (24max). Particularly, hosVIS (24max) had the highest accuracy (AUROC = 0.925, *p* = 0.007, sensitivity = 80%, specificity = 90%). The critical value of hosVIS (24max) for predicting severe outcomes was calculated (cut-off value = 17, Youden index [J] = 0.75).

### Independent risk factors for prognosis

We performed interaction analysis on model variables and determined that there was no interaction between variables. Interaction was omitted from the analytical model. The indicators with p < 0.05 in the univariate analysis were incorporated into the multivariate logistic regression to explore independent risk factors for mortality in neonates with CDH. There was a substantial correlation between the four VIS scores, and there would be multicollinearity when they were incorporated into the multivariate analysis. Since a result, we added the hosVIS (24max) with the highest area under the ROC curve into the multivariate analysis model, as it was deemed to be the most representative. The results revealed that the liver herniation, hosVIS (24max), and postoperative peak lactate level on the first day were independent risk factors for mortality (Table 4).

**Table 4:** Independent risk factors for predicting prognosis

	B	SE	Wald	OR	95% CI	P-value
Liver herniation	2.980	1.530	3.794	19.694	0.982–395.119	0.046
HosVIS (24max)	4.434	1.557	8.108	84.229	3.982–1781.594	0.004
Postoperative lactate level	4.479	1.466	9.339	2.364	4.985–1558.682	0.002

SE, standard error; OR, odds ratio; CI, confidence interval; hosVIS (24max), maximum vasoactive-inotropic score during the first 24 h of hospitalization

## Discussion

We discovered a substantial correlation between VIS and death rates. The VIS was an easily quantifiable measure of support for vasoactivity that was highly linked with the risk of mortality among CDH infants. In this retrospective research, the highest value of HosVIS (24max) was independently related to the mortality of neonates diagnosed with CDH. Even if the other three VIS indicators also have a greater predictive performance, the 24-hour maximum value of the VIS may have a more accurate early predictive performance. The VIS is easy to measure, and the results may be calculated instantaneously at the patient’s bedside. Currently, it is used to assess the severity of cardiac malfunction and to estimate the degree of cardiovascular drug support in infants after cardiac surgery [13]. The cause of CDH-related death is respiratory and circulatory failure [14]. After delivery, the clinical manifestations of CDH in infants are associated with PH and PPHN [15]. It presents first as persistent hypoxia with differential cyanosis [16]; therefore clinical therapy tends to concentrate on respiratory support while ignoring circulatory management. PPHN typically causes early cardiac insufficiency and reduced cardiac output in neonates with CDH. Typically, right heart failure develops first, followed by left heart failure [17]. In neonates with CDH, preoperative cardiac dysfunction often develops within 24–48 h after birth [18]. Nonetheless, more attention should be devoted to the stability of hemodynamics, which was the primary purpose of monitoring VIS in this work.

These patients require inotropic vasoactive assistance. The treatment of infants with CDH should focus on maintaining systemic circulation pressure, which is dependent on vasoactive drugs, because the treatment for reducing pulmonary hypertension cannot take effect immediately [19]. Based on the severity of cardiac malfunction and blood pressure, clinicians will adjust the medication dose and treatment strategy [20]. There is currently no indication to measure the degree of cardiovascular pharmaceutical assistance for children with CDH. VIS contains vasoactive and positive inotropic drugs usually administered to neonates with CDH. Consequently, VIS can not only reflect the severity of PPHN in neonates, but also the difficulty of treating pulmonary hypertension. In this study, VIS may be used to establish the degree of cardiovascular medication support, assess the severity of the disease, and act as an early predictor of mortality in infants with CDH.

Few studies have been conducted on VIS in infants with CDH; our results revealed that liver herniation, hosVIS (24max), and postoperative peak lactate level on the first day were independent risk factors for mortality. In this study, all neonates with CDH were intubated endotracheally and provided mechanical ventilation shortly after birth, and vasoactive medications were administered to 62 neonates in order to maintain their hemodynamics prior to surgery. Consequently, this group of infants were considered to be relatively severe. Therefore, an index was required to evaluate the patient’s hemodynamics at an early stage. According to the results of the investigation, hosVIS (24max) had the greatest accuracy (AUROC = 0.925, p = 0.007, sensitivity = 80%, specificity = 90%). The hosVIS (24max) critical value for predicting severe outcomes was determined (cut-off value = 17, Youden index [J] = 0.75). It has a high level of predictability. Between 2010 and 2019, 15 studies, 9 with pediatric patients and 6 with adult patients, validated the VIS in various scenarios, including cardiac surgery (n = 10), septic shock (n = 3), cardiogenic shock, and traumatic brain injury (n = 2) [21]. The number of identified VIS cut-off indicators of poor outcome ranged from 10 to 30. Notably, the definition of a bad result varied between studies, and none of the studies reported the same VIS cut-off [21]. Our study found that a hosVIS (24max) ≥ 17 was an independent risk factor for death in neonates with CDH.

In clinical practice, it is quite common to combine several vasoactive and pulmonary artery pressure-lowering medicines [22]. Such a balanced strategy is key to successful treatment. The standardization of the treatment scheme for vasoactive medications remains a challenging task due to the fact that vasoactive drug treatment decisions depend extensively on the results of adult confirmatory medical research and the clinical experience of pediatric professionals [21]. Pulmonary artery pressure lowering medications and vasoactive drugs frequently interact during therapy, therefore it is vital to weigh the advantages and negatives, maintain a balanced relationship between drugs, and continually identify the most significant treatment contradictions [23]. This procedure should be repeated.

This study had some drawbacks. The study was conducted as a retrospective study at a single institution, focusing on the institution’s CDH management practices. Institutional policies and practices surrounding the usage and dosage of vasoactive medicines may have an impact on VIS. Neonates with CDH’s cardiac function may be impacted by the mode and circumstances of mechanical ventilation, the use of analgesia and sedation, and other factors, impacting the dosage of vasoactive drugs. Consequently, more samples are required to record the continuous changes of VIS indicator values in order to develop more accurate predictors. Multicenter prospective trials are being prepared to examine the generalizability of our findings. In particular, we intend to use VIS in conjunction with other risk indicators in the future to stratify the risk of neonates with CDH and forecast when extracorporeal membrane oxygenation will be necessary.

## Conclusions

VIS is a substantial independent risk factor related with CDH neonatal death. It has the potential to serve as an early evaluation indication of the severity of pulmonary hypertension and the efficacy of vasoactive medications. The VIS is an inexpensive, noninvasive, accurate, and easy-to-obtain predictor. According to the findings of this research, the cut-off value of HosVIS (24max) for estimating the risk of CDH-related neonatal mortality is 17.

## Data Availability

All data generated or analyzed during this study are included in this article. Further inquiries can be directed to the corresponding author.

## List of abbreviations

AUROC: area under the ROC curve
GA: gestational age
CDH: congenital diaphragmatic hernia
hosVIS (24max): maximum VIS during the first 24 h of hospitalization
hosVIS (24mean): mean VIS during the first 24 h of hospitalization
O/E LHR: observed-to-expected lung-to-head ratio
PH: pulmonary hypertension
PPHN: persistent pulmonary hypertension
postVIS (24max): maximum VIS during the first postoperative 24 h.
postVIS (24mean): mean VIS during the first postoperative 24 h
ROC: receiver operating characteristic
VIS: vasoactive-inotropic score
